# Controlling the Response: Predictive Modeling of a Highly Central, Pathogen-Targeted Core Response Module in Macrophage Activation

**DOI:** 10.1371/journal.pone.0014673

**Published:** 2011-02-14

**Authors:** Jason E. McDermott, Michelle Archuleta, Brian D. Thrall, Joshua N. Adkins, Katrina M. Waters

**Affiliations:** 1 Computational Biology and Bioinformatics, Pacific Northwest National Laboratory, Richland, Washington, United States of America; 2 Biological Sciences Division, Pacific Northwest National Laboratory, Richland, Washington, United States of America; 3 Biological Separations and Mass Spectrometry, Pacific Northwest National Laboratory, Richland, Washington, United States of America; National University of Ireland Galway, Ireland

## Abstract

We have investigated macrophage activation using computational analyses of a compendium of transcriptomic data covering responses to agonists of the TLR pathway, *Salmonella* infection, and manufactured amorphous silica nanoparticle exposure. We inferred regulatory relationship networks using this compendium and discovered that genes with high betweenness centrality, so-called bottlenecks, code for proteins targeted by pathogens. Furthermore, combining a novel set of bioinformatics tools, topological analysis with analysis of differentially expressed genes under the different stimuli, we identified a conserved core response module that is differentially expressed in response to all studied conditions. This module occupies a highly central position in the inferred network and is also enriched in genes preferentially targeted by pathogens. The module includes cytokines, interferon induced genes such as Ifit1 and 2, effectors of inflammation, Cox1 and Oas1 and Oasl2, and transcription factors including AP1, Egr1 and 2 and Mafb. Predictive modeling using a reverse-engineering approach reveals dynamic differences between the responses to each stimulus and predicts the regulatory influences directing this module. We speculate that this module may be an early checkpoint for progression to apoptosis and/or inflammation during macrophage activation.

## Introduction

Macrophages respond to diverse signals when confronted with different challenges and stimuli; from infection with pathogenic bacteria to uptake of inert particles. Collectively these signals form pathogen or damage associated molecular patterns (PAMPs and DAMPs) that convey a wealth of information to the macrophage and drive appropriate innate immune responses [1], [2], [3]. The signatures of intracellular pathogens, PAMPs, elicit a primary response in the host that is largely driven by the Toll-like receptor (TLR) pathway.

Although activation of macrophages through the TLR and other pathways and their downstream regulatory programs are popular topics in immunology [4], [5], [6], [7], [8], [9], the global regulatory responses of the innate immune system are largely unknown. For example, another component of macrophage response is a poorly understood process by which particles of different sizes are recognized. As a model system for particle recognition, manufactured nanoparticles of various kinds elicit a range of regulatory events based on the physico-chemical properties of the nanoparticles and cell type-specific recognition and uptake pathways. We have previously shown that changes in gene expression profiles in macrophages could be directly correlated with particle surface area across a size class distribution of silica nanoparticles [10]. While it is known that nanoparticles stimulate inflammation and induce macrophage cytotoxicity *in vitro* [11], it is not understood how these particles are recognized by the macrophage and how the signaling pathways and transcriptional responses compare to those of the TLR pathway and bacterial recognition.

In contrast to particle recognition and uptake, *Salmonella enterica* serovar Typhimurium *(S.* Typhimurium), an intracellular pathogen secretes a battery of bacterial proteins which are delivered to the host cell [12], [13]. The secreted effectors are known to hijack host cellular machinery and thereby modulate gene regulation. This allows the bacteria to persist and replicate inside the macrophage, an extremely inhospitable environment. Extensive research has lead to the identification of more than 40 secreted virulence factors [12], [14], however, the full function of most remain unknown. In addition, machine learning algorithms suggest that as many as 300 additional proteins may be secreted by *Salmonella* [15]. It becomes apparent that *Salmonella* infection of the host cell is a complex and sophisticated process, one that, unlike the other stimuli, is adaptive and partly driven by the bacteria itself. In fact many pathogens manipulate host responses through direct and indirect interactions between pathogen and host proteins.

Understanding the host immune response requires delving into its complexity and specificity. In this study, we compare gene regulation in response to three different kinds of stimuli: inert particle uptake, TLR agonist treatment, and *Salmonella* infection. The aim of this comparison is to examine and model the commonalities of responses across these various classes of stimuli. We analyze microarray data from a global analysis of gene expression profiles over many types of macrophage challenges including infection with *S*. Typhimurium and two different sizes of amorphous silica nanoparticle (10 nm, and 300 nm). This data set gives us a broad perspective for understanding host response and requires the appropriate bioinformatic analysis to interrogate key regulators of innate immunity. Coexpression networks relate groups of genes together in a network that have similar expression patterns over a range of conditions. Inference of these networks can identify functional modules [16], [17], [18] and provide predictions of regulatory interactions [19], [20]. Though the expression of a gene does not necessarily reflect the activity of its product, the activity of transcription factors or other factors that influence the expression of sets of downstream genes is reflected in the changes in transcription of their targets.

A common task in the analysis of high-throughput data sets is the identification of useful and informative targets that represent hypotheses for further experimental validation. Ideally these targets should be core mediators of important processes and not downstream components of the response [21]. Previously, we have described a novel method to analyze the topology of inferred coexpression networks for identification of potential mediators of system transitions from microarray data and proteomics [22], [23].

In the current study we use a similar approach to identify potential mediators of immune response processes. Our approach is unique in that we are extending existing methods, integrating, and applying them in an effort to more fully elucidate the underlying regulatory network. Combining a network topology approach with comparative analysis of differentially expressed genes, we identify a macrophage core response module that is shared under all conditions. In order to elucidate regulatory influences of the core response module to provide a comprehensive, parsimonious regulatory network we apply a multivariate regression technique. This study provides a number of interesting and novel insights into macrophage response to pathogens, and outlines a valuable and informative set of tools to identify critical nodes in the host response to pathogens.

## Results

### Overview of approach

**Figure 1:**
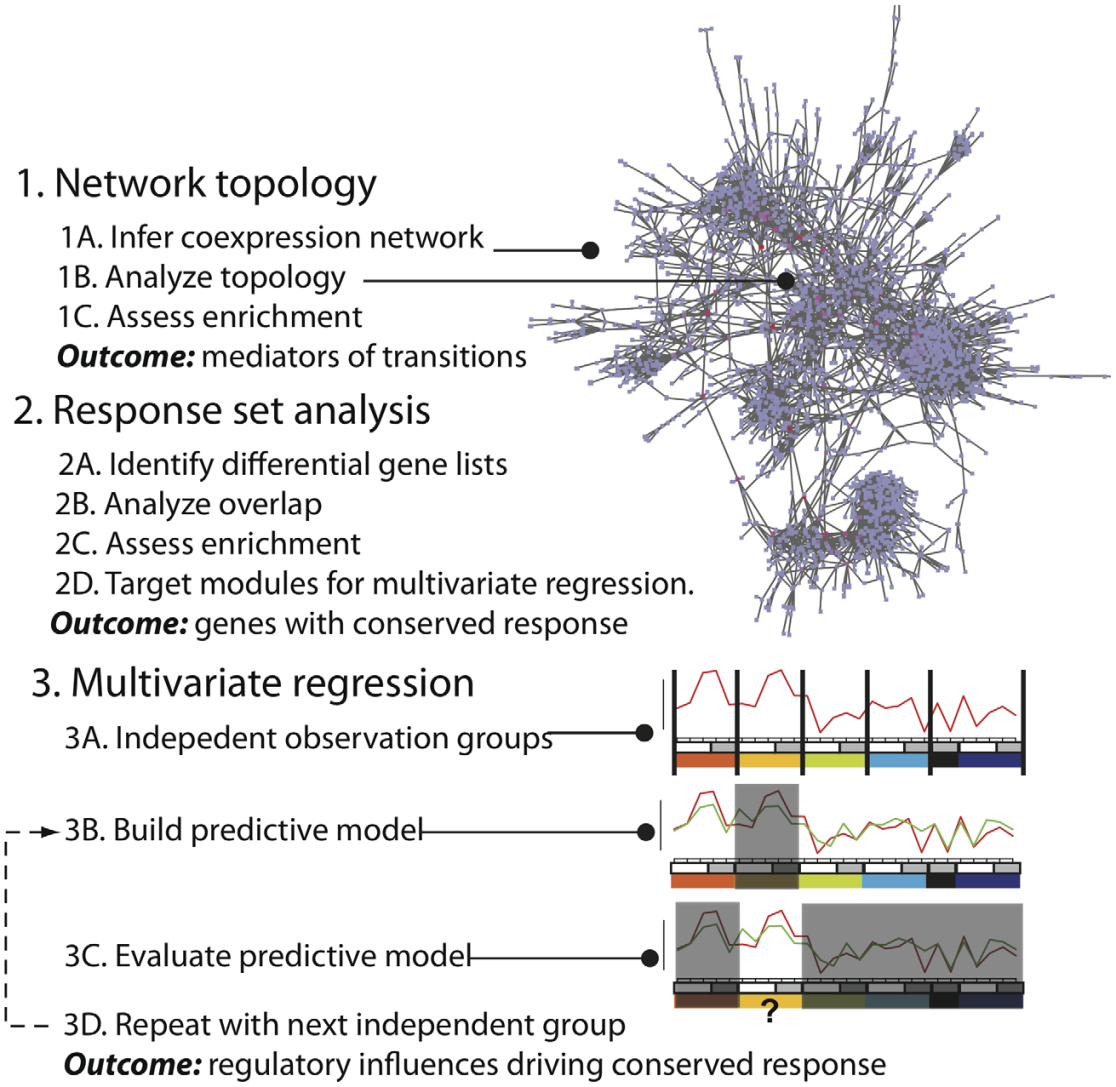
Overview of computational approaches.

Our overall goal in this study was to characterize the similarities between macrophage responses to multiple stimuli, including an intracellular bacteria (*S.* Typhimurium), and inert particles, and to identify important regulatory influences in macrophage activation. To accomplish this we used several different computational approaches (Figure 1). First we inferred regulatory association networks using the Context Likelihood of Relatedness (CLR) method [19]. CLR establishes relationships (edges in a graph) between two genes when the expression of one gene has significant mutual information (i.e. highly similar or dissimilar) with the expression of another gene. The resulting networks summarize the functional dynamics of the system, for the conditions considered. We used this coexpression network to predict important regulatory influences using topological analysis. We then compared the responses of macrophages to a number of important stimuli; TLR agonists, bacterial infection, and inert nanoparticle exposure. We used this analysis to identify a set of genes that was differentially regulated under all conditions examined. To understand the regulation of this core response module we used a multivariate regression method to develop a model of the regulatory influences of the module. This model was validated by assessing its ability to predict gene expression under novel conditions. We finally discuss the results of this analysis in terms of biological insight offered into macrophage activation.

### Network topology identifies important pathogen targets

In order to differentiate the mediators of innate immunity from downstream effectors, we first inferred coexpression networks from a compendium of high-throughput datasets examining macrophage response (as described in Methods). Applying the CLR method [19], we established significant relationships between genes, being defined with Z scores greater than four. Then we determined the topology of these relationships by identifying the number of neighbors a gene has in the network (degree) and the role the gene plays as a linker to bridge disparate regions of the network (betweenness). Genes with the highest betweenness and degree values are defined as bottlenecks and hubs, respectively.

To determine the significance of the topology in the inferred networks we compared betweenness values from inferred and randomly rewired networks. We ranked genes based on betweenness values and compared the difference between the betweenness values for the real network and mean betweenness from 100 randomized networks for the same rank. This analysis (Figure S1) showed that the real networks have very different topologies than randomized networks, and betweenness values for the real networks are much larger than in the randomized networks (Z scores>20). This was observed even when a very small percentage of the edges are reassigned, showing that even small changes to the network change the topology.

**Figure 2:**
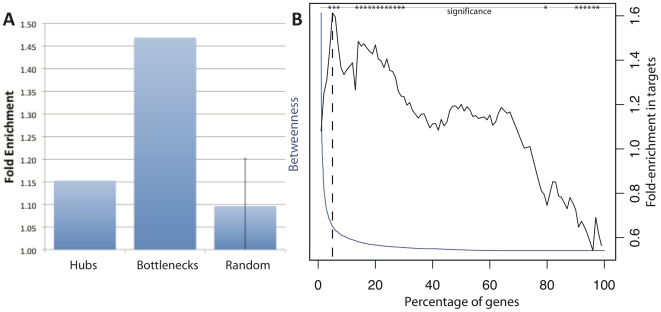
Topological bottlenecks in inferred networks are enriched in pathogen targets. **A.** Hubs and bottlenecks (top 20% of degree and betweenness values, respectively) were analyzed for their enrichment in known targets of pathogens (blue bars). Bottlenecks are significantly enriched (p-value 0.004) in pathogen targets, but not human homologs, and hubs were enriched in neither. Additionally, the mean enrichment of bottlenecks from 100 randomized networks is shown, with error bars representing +/− one standard deviation. **B.** Bottlenecks were identified using between 1 and 100% of the top ranked betweenness values in the network (x axis) and the enrichment in pathogen targets versus non-bottlenecks is shown (black line). The betweenness values are shown as a blue line. Significant fold change values (p-value<0.05) are indicated by asterisks at the top of the figure. The dotted line indicates the location of the peak of greatest enrichment. These results indicate that bottlenecks from inferred networks are more important to the functioning of the system than other genes.

Based on the previous observations from our group [22], [23] and others [24], [25], we believed that highly central genes in these inferred networks (hubs and bottlenecks) would be more biologically relevant to the system. We therefore assessed the enrichment of hubs and bottlenecks in conserved genes and genes that code for proteins known to be targeted by pathogens [24]. In Figure 2A, we show the fold enrichment in pathogen targets for hubs, bottlenecks and bottlenecks derived from randomly rewired networks (random bottlenecks) versus other genes in the network. These results show that hubs and bottlenecks in random networks were not enriched in pathogen targets but that bottlenecks were significantly enriched (p-value 0.004) in pathogen targets in the real network. Similar to previous observations from protein-protein interaction networks [24], these results indicate that genes with high betweenness centrality in inferred networks are more likely to be targeted by pathogens, thus probably play important roles in the response to pathogens. We found no significant enrichment in homologs in either hubs or bottlenecks (data not shown).

The results in Figure 2A show that genes with the top 20% of betweenness in the network are significantly enriched in targets of pathogens but we were interested in determining if network betweenness correlated with the probability that a gene is a known pathogen target. We therefore varied the threshold we used to classify a gene as a bottleneck (Figure 2B). By increasing the threshold, more nodes were classified as bottlenecks (x-axis) and their betweenness values showed a rapid decline (blue line) in the top 5% of values. This ‘elbow’ (indicated by the vertical dotted line) indicates that there are two populations of genes in the network; a small number with exceptionally high betweenness values and a large number with low betweenness values. This is corroborated by our analysis of the distributions of betweenness values relative to random networks (Figure S1). The fold enrichment in pathogen targets displays a poor overall correlation with betweenness only showing significant enrichment in the top 20–25% of genes. However, the maximum fold enrichment in pathogen targets (black line) occurs when the top 5% of genes are classified as bottlenecks. This indicates that there is a small population (top 1–5%) of evolutionarily conserved genes with exceptionally high levels of betweenness in the network, which may be global regulators of information flow, an idea supported by our analysis of the distribution of betweenness values (Figure S1). The secondary peak at 15–25% may represent another population of bottlenecks.

### Comparative analysis between conditions identifies the core response module

**Figure 3:**
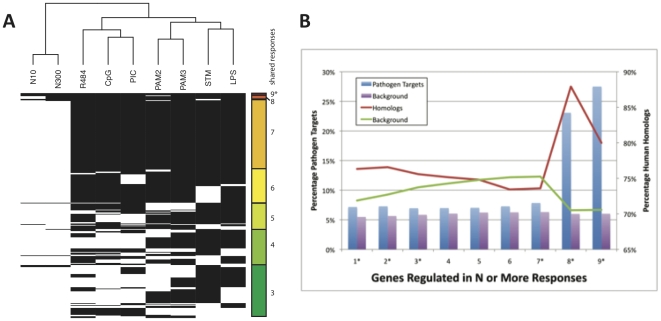
Response set analysis in macrophages. **A.** Genes (rows) with shared differential expression in response to multiple stimuli (columns) are shown with black boxes indicating differential expression. The plot is ordered from genes differentially regulated in all conditions examined (9*, the core response module), to those differentially regulated in three conditions (bottom). A dendrogram showing the similarity between stimuli is shown at top; N10, 10 nm nanoparticle; N300, 300 nm nanoparticle; STM, *Salmonella* infection. **B.** The percentage of pathogen targets (bars) in each group of genes (blue bars) or in background (not in the group; purple bars) is shown for each group of genes regulated by N or more stimuli (X axis). The corresponding analysis is shown for Human homologs (lines) for the group (red line) or background (green line) in each group. Asterisks by each group on the X axis indicates that these groups are statistically enriched in both homologs and pathogen targets, other values were statistically significant after multiple hypothesis correction. These results show that groups of genes that are differentially regulated in response to a broad range of stimuli are more likely to be targets of pathogens and are more conserved than other genes.

The broad spectrum of stimuli in our data set gave us the opportunity to identify the essential conserved components of macrophage activation. Differentially regulated genes were identified using a 1.5 fold expression change threshold for probes that passed a significance test up to 360 minutes post-treatment. We observed that the responses of macrophages to nanoparticles were delayed relative to the other stimuli, and that very few differentially expressed genes overlapped with the compendium; therefore, we considered the entire time course, up to 24 hours post-treatment. To elucidate the components of macrophage activation, we identified groups of genes that are regulated by different numbers of conditions (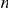). The results of this analysis are shown in Figure 3A, where black indicates that genes (rows) are differentially regulated in a given response (columns). Table S1 provides the complete list of genes, the conditions under which we found differential regulation, the network properties of the gene, and its status as a pathogen target.

Our results show that amorphous silica nanoparticle exposure is quite different from the other stimuli, and appears to elicit a much milder response when compared with the other conditions studied. We observe a division between typically viral-like (R848 and PIC)/shared (CpG) stimuli and bacterial-like (LPS, PAM2 and PAM3) stimuli, with *Salmonella* (STM) showing similar patterns of gene regulation as the other bacterial stimuli. The genes are ordered according to their 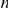, number of shared response as shown in the legend on the right hand side. We identified a subset of 38 genes are differentially regulated by all nine conditions (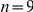) examined within the data set and call this group (Figure 3A, top rows) the macrophage ‘core response module’. To assess the significance of this result we performed 10,000 random selections and found that in no case did this yeild an overlap of even one gene, indicating that the p-value for finding 38 matching genes is well below 0.001. A selection of the more interesting members (bottlenecks and/or pathogen targets) are listed in Table 1 and the full list of genes with associated information is provided as Table S1.

**Table 1:** Members of the macrophage core response module.

Symbol	Description	Bottleneck	Target	Function
Ccl3	chemokine (C-C motif) ligand 3	5%	Yes	IM
Ccl4	chemokine (C-C motif) ligand 4		Yes	IM
Cxcl2	chemokine (C-X-C motif) ligand 2	5%		IM
Egr1/2	early growth response 1 and 2		Yes	TF
Fdft1	farnesyl diphosphate farnesyl transferase 1	5%		
Fos	FBJ osteosarcoma oncogene		Yes	TF
Gadd45b	growth arrest and DNA-damage-inducible 45 beta			ST
Ifi44	interferon-induced protein 44	20%	Yes	IM
Ifih1	interferon induced with helicase C domain 1	20%	Yes	IM
Ifit1/2	interferon-induced protein with tetratricopeptide rep. 1/2	10%		IM
Jun	Jun oncogene	5%	Yes	TF
Mafb	v-maf musculoaponeurotic fibrosarcoma oncogene family	5%		TF
Mx1/2	myxovirus (influenza virus) resistance 1 and 2		Yes	IM
Oas2	2'-5' oligoadenylate synthetase 2		Yes	IM
Oasl1	2'-5' oligoadenylate synthetase-like 1			IM
Osgin2	oxidative stress induced growth inhibitor family member 2	20%		ST
Plau	plasminogen activator, urokinase		Yes	
Ptgs1	prostaglandin-endoperoxide synthase 1 (Cox-1)			IM

Bottleneck, the approximate level of betweenness for genes in the top 20%; Target, if product of the gene is identified as a known pathogen target; Function, general functional group (IM, immune function; ST, stress response; TF, transcription factor). Genes not listed: B230342M21Rik, BC013672, LOC545174, Ddit3, Edg1, Gadd45b, Gbp3, Irgm, Klf6, Ms4a6b, Mthfd2, Parp12, Plau, Rnd2, Sc4mol, Scd1, Sesn2, Slfn4, Tyki. All genes considered are listed in Table S1.

In addition, we looked at members of the core response module to determine their importance in the topology of the network. We found that the core response module is 2.5 fold enriched in bottlenecks (p-value 1.74E-05), and that the module is highly central in the network (p-value 2E-16 by t test). Interestingly AP1 (Fos and Jun) and Egr1 and 2 were among the list of bottlenecks within the core response module, regulators which are known to be important for early macrophage response [26], [27]. This result is consistent with the idea that the members of the core response module might be more significant to the functioning of the system, as indicated by their regulation in response to many different stimuli.

To further determine the importance of genes with conserved responses to multiple stimuli we assessed the fold enrichment of pathogen targets (bar graph Figure 3B) and human homologs (lines Figure 3B) for 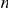, number of overlapping conditions. While there was minimal enrichment in pathogen targets and homologs when compared to background levels for conditions, 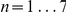; there was significant enrichment in both pathogen targets and homologs for 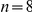 and the core response module (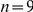). These results show that the core response module has 80–90% conservation with human homologs (1.25 fold enrichment above a background of 70%, Bonferroni adjusted p-value 1.0E-03) and is comprised of 23–28% pathogen targets (4.5 fold enrichment above a background of 6%, Bonferroni adjusted p-value 7.5E-07). Groups of genes shared in fewer numbers of conditions do not show a high degree of enrichment or an increasing trend. These results strongly support the notion that the core response module is playing an important and conserved role in macrophage activation, one that is preferentially targeted by a range of pathogens and is enacted by evolutionarily conserved genes.

To examine the importance of the core response module in known pathways of macrophage activation we used a curated set of macrophage protein-protein interactions [26]. We found that 29 gene products from the highly conserved gene set (differential expression in 8 or 9 stimuli) were involved in known interactions, versus 390 gene products from the remainder of the genes (6.9%). The expected ratio is given by the ratio of the total numbers of genes in each group (92 versus 7414; 1.2%), and this gives a p-value of less than 0.0001. So the highly conserved response is more likely to participate in interactions important for macrophage activation.

To assess the contribution of the nanoparticle response to the core response module the functional enrichment in gene ontology categories in the highly conserved set of genes (those genes differentially regulated in 8 or 9 conditions) was examined relative to the genes that were conserved in all conditions except in response to nanoparticle exposure. This analysis showed that genes with a universally conserved response were enriched in cell cycle processes (p-value 5E-4) and anti-apoptosis (p-value 2E-3). Since nanoparticle exposure and infection with *Salmonella* share the characteristic of being exposure to particles we calculated the enrichment of genes differentially expressed in response to both nanoparticles and *Salmonella* infection (particulate response) to those differentially expressed in response to all TLR agonist treatments (non-particulate response). We found that the particulate response was significantly enriched in isoprenoid biosynthetic processes (p-value 4.4E-5), cholesterol biosynthesis (p-value 2.1E-4), and cell differentiation (p-value 2.7E-4). Cholesterol and other lipids are known to play roles in macrophage response to *Salmonella* infection. The *Salmonella* containing vacuole, a membrane bound compartment in which Salmonella resides intracellularly, recruits up to 30% of cellular cholesterol during infection [28] and the *Salmonella* secreted effector SseJ is targeted at cholesterol esterification, which is important for bacterial survival [29]. Our findings suggest that some aspects of lipid metabolism response in macrophages may be modulated as part of a specific particle response, as opposed to through TLR pathways.

### Dynamic regulation of the core response module

**Figure 4:**
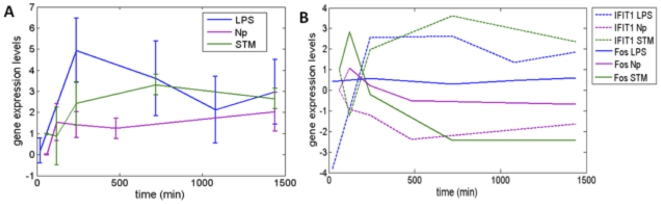
Dynamics of core response module. A) Temporal regulation of gene expression levels for a cluster of upregulated genes in the core response module. The three conditions LPS, Np (nanoparticle), and Salmonella (STM) are labeled with blue, purple, and green lines. Error bars signify 95% confidence and the average is over all gene expression profiles within a cluster. B) Individual gene expression levels of Ifit1 (dashed line) and Fos (solid line) under each condition LPS (blue), Np (purple), and STM (green).

In order to properly assess the core response module it was essential to determine the dynamics of gene regulation. We examined the core response module, focusing in Figure 4 on a subset a set of upregulated genes. To gain further insight into the dynamics of the core response module in *Salmonella* infection, which has a limited number of observations in mouse macrophages, we compared expression in another independent dataset: human macrophages infected with *Salmonella* [30]. Figure 4A shows the dynamics of the upregulated genes within the core response module: for each of the conditions LPS (blue) and nanoparticle (purple) treatment, and *Salmonella* infection (green). We observed differences in the timing and magnitude of response; LPS elicits a more pronounced response than either the *Salmonella* or nanoparticle exposure. Comparing *Salmonella* and LPS we observe a more delayed and less amplified gene response in *Salmonella*. We speculate that the lag time in *Salmonella* could be an attribute of *Salmonella* secreted effectors modulating members of the core response module. The dynamics of nanoparticle exposure appears to elicit a much milder response than either *Salmonella* or LPS.

We next looked at Ifit1, and Fos, which have been identified as bottlenecks and are known (Fos) [26] or predicted to be (Ifit1) central regulators of innate immunity. Ifit1 showed a similar sustained response when simulated with both LPS and *Salmonella* exposure. Nanoparticle stimulus appeared to inhibit Ifit1 expression levels. In contrast, *Salmonella* appears to induce a rapid response in Fos expression that is quickly downregulated, whereas LPS induced a moderate response. Fos responding to nanoparticle exposure shows a similar initial trend as in *Salmonella* infection but has a less dramatic drop off. The implication of these findings suggests that *Salmonella* may be directly or indirectly altering Fos gene expression level.

### Regulatory influences driving the core response module

Network inference using CLR and topological analysis provided some information about the potential regulation of the core response module, but did not provide detailed information about regulatory influences. Thus, to predict causative regulatory influences acting on the core response module, we applied a multivariate regression approach, previously developed and used in microbial systems [31]. This approach uses L_1_ regression [32] to learn a parsimonious set of regulatory influences that best describes the behavior of a target cluster. Using only the expression levels of the inferred regulators the inferred model can predict the dynamics of the target at future time points. In addition, the resulting model can be used to evaluate the transcriptomic behavior of the target cluster under novel conditions. The coexpression networks inferred by CLR above provide valuable information about the general associations between genes and functions. However the multivariate regression approach employed provides additional insight into the regulatory network by prediction of the directionality of regulatory interactions. Importantly, this approach allows quantitative prediction of the influence of regulators on their targets under novel conditions, which can be used to validate these predictions.

The approach is based on a number of assumptions. The first is that the mRNA abundance levels reflect the activity of the regulator it encodes. Therefore the approach can only be successful in the cases where this assumption is met, or in a case where the activity of a regulator is reflected by the expression levels of another closely associated gene. A second assumption is that clustering applied to the expression data will identify co-regulated groups of genes, or, alternately, that it captures important trends in the data that may arise from multiple influences. The resulting models can be validated empirically by assessing how well they can explain the expression of the prediction target clusters under conditions not used to parameterize the model. Thus, the models arising from this approach provide a useful basis for making predictions and can be validated computationally, but are unlikely to capture all the details of the system in a complete and fully accurate manner.

**Figure 5:**
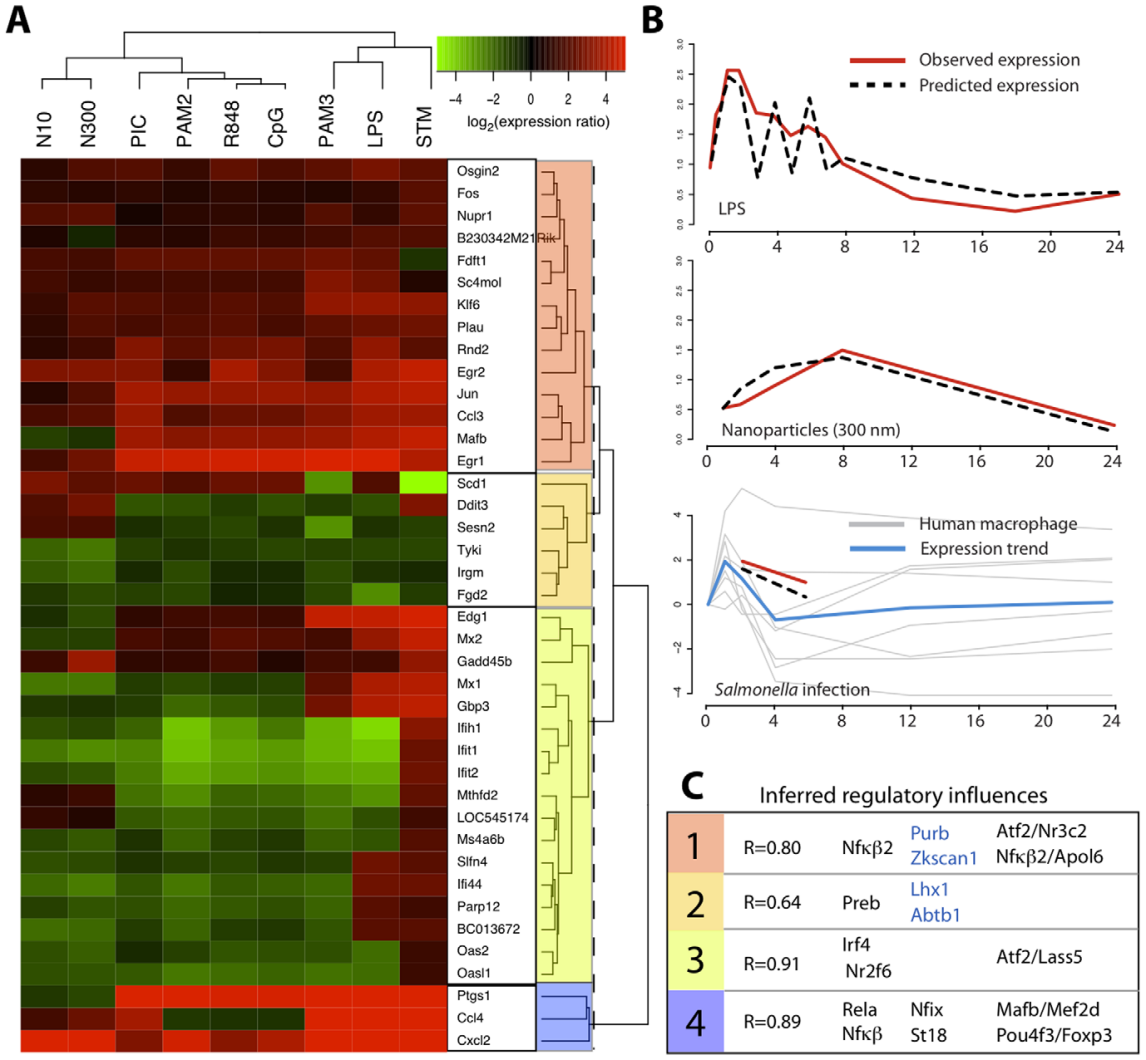
Modeling the dynamics of the core response module. **A.** Heatmap representation of the expression of the core response module. Each row represents a gene and each column represents a time series. The values in the heatmap are the maximum absolute value of differential expression from all time points. Shown at right is a dendrogram indicating the relationships between the genes and the color bars indicate sub-clusters that were used for further modeling. **B. Predictive dynamics of regulatory cluster.** The observed (red lines) versus predicted (dashed black lines) expression for cluster 1 (the regulatory cluster) is shown over a 24 hour time period after exposure to LPS or 300 nm nanoparticles, or infection with Salmonella. Given the sparse *Salmonella* infection data in mouse macrophages we use the expression of genes (grey lines) from the cluster in a study of infection of human macrophages to illustrate cluster dynamics. The mean expression of the genes is shown as a blue line. **C. Inferred regulatory influences for core response sub-clusters.** The correlation of predicted to observed expression is listed for each sub-cluster. Predicted regulators for the cluster are listed; black indicates a positive influence, blue indicates a negative influence, and pairs of regulators separated by a slash denote inferred combinatorial influences.

Using hierarchical clustering, we divided the core response module into four subclusters as shown in Figure 5A, to represent potentially distinct regulatory programs responsible for the transcription of the core response module. These subclusters are enriched in transcription factors (red), genes with no known role in macrophage activation (orange), interferon regulated factors (green), and inflammation (blue). They are clustered vertically according to condition. For modeling the subclusters are used as targets and potential regulators are a list of 331 differentially expressed genes annotated as transcription factors by gene ontology [33]. Using both targets (clustered gene expression data) and regulators we performed cross-validation; wherein, each set of measurements for a given condition (i.e. LPS) is left out of the training set and used to evaluate the performance of the resulting inferred network. The results from each independent evaluation are scored using Pearson correlation between predicted and observed expression for each subcluster. The results of cross-validation yield a high gene-normalized average correlation of 0.83 over all 25 independent condition groups in the macrophage compendium. We then used the inferred regulatory model derived from subcluster 1 to predict the dynamic gene expression of the core response module under LPS and *Salmonella* infection, as shown in Figure 5B. The predicted expression (dashed line, panel 1) closely approximates the observed (red line, panel 1) dynamic gene expression for LPS; showing an initial amplification followed by gradual decline. To validate the model we applied it to predict the subcluster expression in the nanoparticle data, which is a very different response (e.g. Figure 3A) from the others and was not used in the cross-validation. Panel 2 (Figure 5B) shows that the model captures the trend of the observed nanoparticle gene expression well.

We tested the regulatory network on the *Salmonella* infection data, including data from *Salmonella* infection of human macrophages [30] to show the dynamics of this cluster. The blue line shows the mean gene expression for the human macrophage data and the gray lines represent the dynamics of single genes. Figure 5C summarizes our results, showing the correlation values using cross validation for regulatory networks derived from subcluster 1 (red), 2 (orange), 3 (green), and 4 (blue). The regulators shown within each subcluster are predicted to be the mediators of gene expression. We observe good overall prediction in networks derived from subclusters 1, 3 and 4 that contain transcription factors, interferon-regulated factors, and components of the inflammatory response, respectively. Subcluster 2 contains several genes with no known role in macrophage activation, presenting a number of interesting hypotheses for further validation, but apparently result from a regulatory program that is not easily predictable by the model. Thus the predictions of regulatory influences for subcluster 2 (listed in Figure 5) are unlikely to be complete, and may represent false positive predictions.

## Discussion

Understanding the mediators of innate immunity requires interrogating compendia of knowledge generated from high-throughput technologies [30]. In this study we analyzed macrophage activation across a broad-spectrum of innate immunity stimuli; inert nanoparticle exposure, TLR agonists, and *Salmonella* infection. Using a combination of bioinformatics techniques we determined a highly focused group of candidate genes for further experimental investigation. The topology of the inferred macrophage regulatory network was used to identify many of these important genes. We showed that the bottlenecks of the network are significantly enriched in known targets of pathogens. This finding can be compared to those reported in the human protein-protein interaction network [24] and our previous findings that bottlenecks were enriched in virulence essential genes [22].

We next examined genes that are differentially regulated in response to multiple stimuli and identified a subset of genes that are differentially expressed under all conditions examined and occupy a highly central location in the inferred network. Interestingly these genes are highly enriched in conserved homologs and pathogen targets, indicating that they are biologically significant in macrophage activation. This group, the macrophage core response module, which encompasses 38 genes including chemotactic cytokines (Ccl3, Ccl4, Cxcl2), transcription factors (Fos/Jun AP-1 complex, Egr1 and 2, and Mafb), apoptosis (Ddit3 and Gadd45b), steroid biosynthesis (Fdft1, Sc4mol, and Scd1), other immune response-related genes (Ifit1, Ifit2, Mx1, Mx2, Oas2, Oasl1 and Ptsg1 [Cox1]) and a number of genes with unknown roles in immune response (see Table 1). Previously, Ramsey, et al. (2008) [27] analyzed a macrophage compendium (also used as part of the present study) using a variety of approaches and described two ‘core early response’ clusters that overlap with our module significantly (∼50% shared genes). Furthermore, through motif enrichment they found that genes in these clusters were enriched in AP1, JUN, CREB, ATF, EGR, and PPARA binding sites, indicating that components of our core response module may be regulated by the Fos/Jun AP1 complex, and the Egr1 and 2 transcription factors that are in the module as well.

To represent the dynamics of the genes in this module and predict the regulatory influences governing their expression we developed a predictive model. By describing the regulatory network of the core response module in a machine learning algorithm [31], we were able to predict gene expression on a new data set. Multivariate regression techniques have been applied to model data in prokaryotes [34] or yeast [35], and here we have successfully applied this method to model data from a mammalian system. Our resulting model accurately predicts the behavior of the core response module in combinations of treatments and genetic backgrounds in a cross-validation approach. Furthermore, the model can accurately predict the expression of a subset of these genes in macrophages responding to nanoparticle exposure, which induces a very different response than the TLR pathway.

Our predictive model identifies a number of regulatory influences that provide the basis for further experimental investigation. Core response module subcluster 1, which is enriched in transcription factors like Egr1/2 and Fos/Jun, is predicted to be regulated by Nfkß2, a component of the alternative Nfkß pathway [36], and negatively regulated by Purb and Zkscan1, intriguingly neither of which has a demonstrated role in innate immunity. Using the Metacore program (GeneGO, St. Joseph, MI) that has a curated database of known regulatory relationships, we found that six of the 14 members of subcluster 1 were known to be regulated by one or more of the regulators inferred in our analysis. Subcluster 3 is composed of many interferon regulated genes, and is predicted to be regulated by Irf4 and Nr2f6. Irf4 may be involved in alternative macrophage activation by IL-4 [37], and is known to regulate Ifit2 [38], but the function of Nr2f6 in macrophages is unknown. Interestingly, ISGF3, a regulatory complex composed of Stat1, Stat2, and Irf9, is known to be a primary regulator of the interferon response, but is not identified by our analysis. This is likely due to the fact that the activity of this complex is not closely tied to the expression levels of its component genes, requiring phosphorylation and assembly of the protein complex itself. This limitation does not refute the predictions made by our approach since it has been shown that regulation of the interferon response is complicated and involves multiple redundant pathways [39]. Finally, cluster 4 is composed of three genes, Ptgs1 (Cox-1), and the cytokines Ccl4 and Cxcl2. These genes are highly upregulated under nearly all stimuli examined and are predicted in our model to be regulated by Nfkß and Rela, the complex responsible for primary activation of the inflammatory response. Strikingly, all three of these genes are known to be regulated by Nfkß, supporting our inferred model. Of the other predicted regulatory influences Nfix has no known immune response functions, but St18 is a known regulator of the proapoptotic response [40], which is related to inflammation.

Our identification and characterization of the core response module suggests that it plays an important role in macrophage activation. The known functions of some of its members, for example AP1, the module's central location in the inferred network, and its preferential targeting by pathogens suggest that it may be an early mediator of downstream functions, possibly as a checkpoint of progression to apoptosis or inflammation. Our analysis suggests that lipid and cholesterol biosynthesis pathways are an important response, a portion of which is triggered by a general response to particles and possibly not through classical TLR pathways, though further investigation is needed to confirm this observation. A future direction is to investigate the downstream functions that the module may be involved in regulating and determine how pathogen proteins may alter this regulation to promote virulence.

Bioinformatic studies of macrophage response to TLR agonists and to bacterial infection using a compendia of transcriptomic data have been published previously [27], [30], and have reported similar core response sets of genes that are much larger than ours. Our study is the first to compare these responses with those elicited by inert manufactured nanoparticles; deducing a more concentrated subset of regulators. We identified lipid and cholesterol biosynthesis pathways as being potentially responsive to particles including nanoparticles and live bacteria. The core response module appears to be highly relevant to macrophage activation as we showed by training on TLR agonist and *Salmonella* infection and very accurately predicting the dynamic behavior of gene expression under nanoparticle exposure. This analysis is significant because it shows that although much of the macrophage response differs for nanoparticles, a set of genes is regulated by all three kinds of responses, and this set, our core response module, seems to be a very important component of macrophage activation.

## Methods

### Datasets

We used three transcriptomic datasets in this analysis. A compendium of 170 microarrays analyzing murine macrophages in time course responses to various stimuli including TLR agonists and *Salmonella* infection, described in greater detail in [27] was used for the CLR network analysis and topology. A compendium of human responses to many different pathogens described in [24] was used to provide dynamics of the core response module to *Salmonella* infection. And the nanoparticle response data is from our study, described below.

Nanoparticle exposure was assessed as follows. The RAW 264.7 murine macrophage cell line was obtained from the American Type Culture Collection (Rockville, MD) and cultured as we have previously described [10]. RAW 264.7 cells were plated in 60 mm plates (7.5×10^5^ cells) overnight and then exposed to 10 nm (5 µg/ml) or 300 nm (150 µg/ml) amorphous silica particles for 1, 2, 4, 8, or 24 hr in serum-free medium. The concentrations used were chosen such that the total administered surface area was the same for both particles sizes and should provide relatively equivalent response pathways, as we have shown previously [10]. Whole genome microarray analysis was performed using Affymetrix Mouse Genome 430A 2.0 chips (Affymetrix, Santa Clara, CA, USA; 22,690 probesets). Raw intensity data were quantile normalized [41] and subjected to analysis of variance (ANOVA) [42] with Tukey's posthoc test and 5% false discovery rate calculation [43] to identify differentially expressed genes. The list of known pathogen targets was obtained from the supplemental data for [24]. Mouse gene ontology annotations and homology relationships were obtained from the MGI [33].

A list of proteins targeted by pathogens was obtained from Supplemental Data in Dyer, et al. [24]. This list was compiled from existing literature and a number of high-throughput screens. This list contains 15,524 physical interactions between pathogen proteins and host proteins including 1234 host proteins and 718 pathogen proteins. In this list, 1134 proteins were found to be targeted by viruses and 124 by bacteria (24 are targeted by both). This bias is largely due to the fact that it is often easier to study the interactions of viral proteins but is not expected to significantly affect the results presented here.

### Network inference and topology

We inferred regulatory influence relationships between genes in the murine macrophage compendium using the context likelihood of relatedness (CLR) method [19]. CLR calculates mutual information between pairs of gene expression profiles then filters the resulting matrix to retain statistically significant relationships between genes.

Network topology measures (degree and betweenness) were calculated using the igraph network library (http://igraph.sourceforge.net/) in the R statistical language. Random networks were generated by transforming the original network using the rewire.edges function with a probability of 0.5 (i.e. half of the edges are randomly reassigned in each network). The values for random networks were obtained as the mean and standard deviation from analysis of 100 random networks.

### Response set analysis

For the response set analysis we used a threshold of 1.5 fold change from control condition (defined according to the particular stimulus). We considered a stimulus to differentially regulate a gene if that gene was greater than 1.5 fold up- or down- regulated by the stimulus at any time point considered in the analysis. Time points for the TLR agonist and *Salmonella* infection time courses were considered out to 6 hours post-treatment, two time points for three strains of *Salmonella* tested, and varying numbers of time points for the TLR agonists. The nanoparticle data set was measured at all time points to 24 hours. To assess the significance of overlap in the fully overlapping set we randomly chose genes from the total 9707 genes considered in sets corresponding to the size of the differentially regulated set for each stimulus. We repeated this process 10,000 times and assessed the number of random genes shared by all stimuli.

### Inference of predictive regulatory models

We used a multivariate regression approach, the Inferelator [31], to infer predictive models based on the transcriptomic dataset. We identified subclusters of the core response module with similar expression patterns using hierarchical clustering (Euclidean distance, complete linkage) and chose to divide the core response module into four subclusters based on visual observation of the cluster dendrogram (Figure 5A). The mean expression of all genes in a target cluster was used as the input to Inferelator. Potential regulators were identified as all genes annotated with the GO category ‘transcription factor activity’.

In the learned model the relation between the expression of a target (y) and the expression levels of regulators with non-null influences on y (X) is expressed as: 
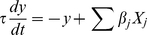
(1)


Here, 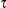 is the time step used in model construction and ß is the weight for relationship X on y as determined by L_1_ shrinkage using least angle regression [44]. To make predictions using a learned model eq. 1 can be solved for y, the expression of the target cluster. Assuming equilibrium conditions the derivative dy/dt is 0 and so equation (1) can be represented simply as a linear weighted sum:
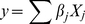
(2)and the dynamic version (for time series) is expressed for each time point (m) as:
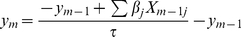
(3)


In our modeling we used a 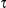 of 30 minutes, which is appropriate for mRNA dynamics in a eukaryote [45].

For determination of regulatory influences we considered only regulators with expression patterns that were correlated with the target at levels below 0.9. This threshold was used to reduce the number of predicted regulatory influences that are based on correlation, but are not true causative influences.

Cross-validation was performed by constructing 25 models, each leaving out a specific set of conditions that reflect a particular treatment (all LPS time points, e.g.) for a total of 25 sets of conditions from the 170 measurements. The resulting model was used to predict the expression of the targets given the expression levels of the inferred regulators. Performance for each target cluster was evaluated using Pearson correlation coefficient between the predicted and observed expression levels for all 170 conditions. Performance is evaluated as the average correlation of observed versus predicted expression values for each target weighted by the number of genes in each target, to produce a weighted gene-normalized overall performance score for the model, as:
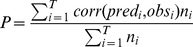
(4)where *P* is the overall performance score, *T* is the number of targets in the model, *pred* and *obs* are the predicted and observed expression patterns, respectively, and *n* is the number genes in the target *i*. This cross-validation approach allows relatively unbiased assessment of model performance because the data used to evaluate the model is not included in the training data.

Regulatory influences were determined by considering those regulators and combinations of regulators present in more than 50% of all independent models from cross-validation. Cross-validation and following analysis was performed using in-house software written in R and available upon request.

### Enrichment

Statistical significance for enrichment in pathogen targets and homologs was calculated using Fisher's exact test and a significance threshold of 0.05. P-values were adjusted using a Bonferroni multiple hypothesis correction, where appropriate.

## Supporting Information

Figure S1 Significance analysis of betweenness values in the CLR-inferred macrophage network. Z scores (Y axes) were calculated for the real betweenness values versus the mean betweenness of the node with the same betweenness rank (X axes) in 100 networks with 50% or 0.1% of the edges rewired. The results show that the betweenness values in real inferred networks are very different from those in randomized networks, even when the networks have been perturbed very little.
(0.60 MB TIF)

Table S1 List of genes considered in network inference and response set analysis. Response group analysis, a 1 indicates that the gene was found to be differentially regulated (fold change 1.5) in the specified stimulus.
(1.38 MB XLSX)
