# Supplementary figures and images for: Controlling the Response: Predictive Modeling of a Highly Central, Pathogen-Targeted Core Response Module in Macrophage Activation
Source: PLoS One. 2011 Feb 14;6(2):e14673. doi: 10.1371/journal.pone.0014673 (PMC3038849; doi:10.1371/journal.pone.0014673)

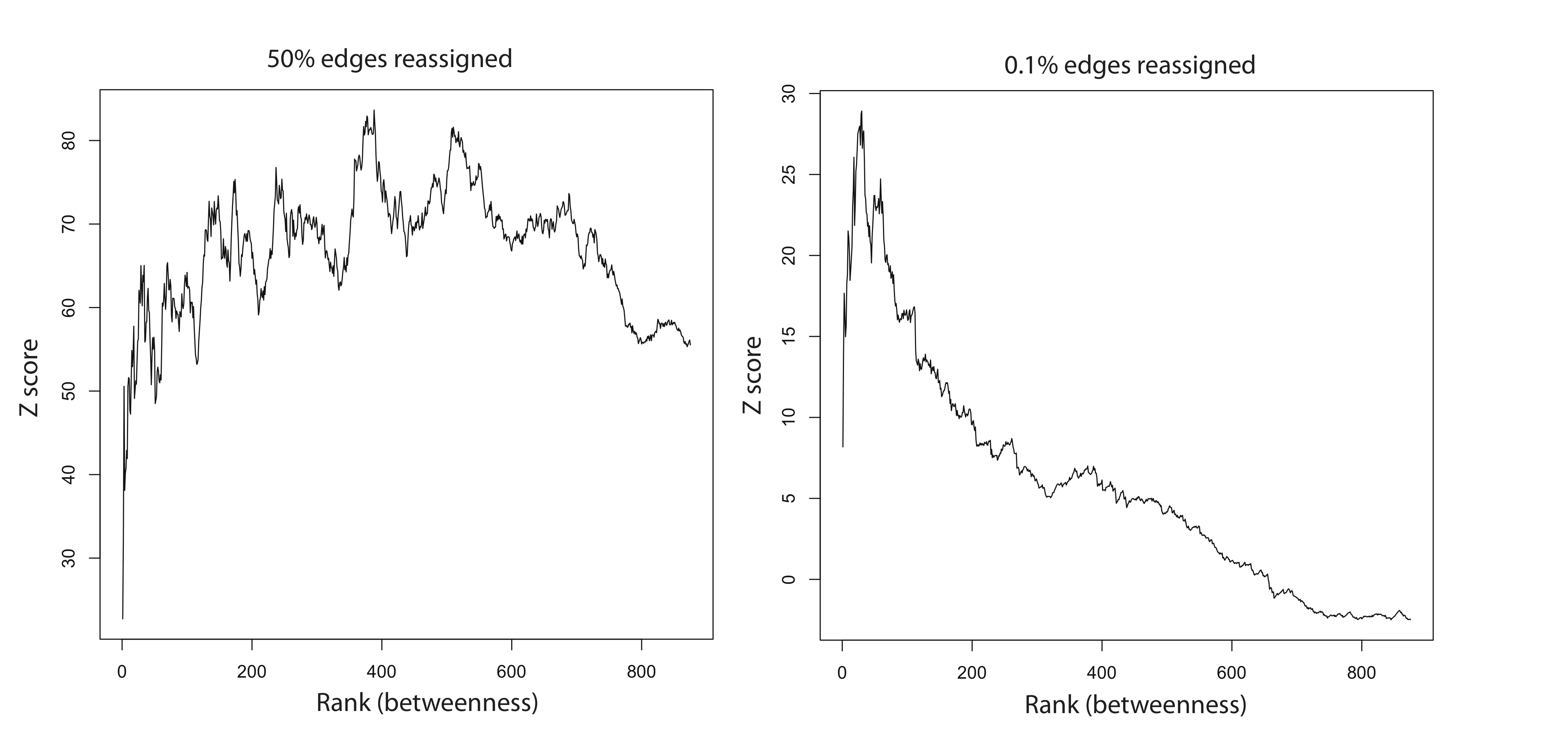

Supplement: Figure S1 — Significance analysis of betweenness values in the CLR-inferred macrophage network. Z scores (Y axes) were calculated for the real betweenness values versus the mean betweenness of the node with the same betweenness rank (X axes) in 100 networks with 50% or 0.1% of the edges rewired. The results show that the betweenness values in real inferred networks are very different from those in randomized networks, even when the networks have been perturbed very little. (0.60 MB TIF) [file pone.0014673.s001.tif]
